# Multitasking muscle: engineering iPSC-derived myogenic progenitors to do more

**DOI:** 10.3389/fcell.2024.1526635

**Published:** 2025-01-22

**Authors:** Mark Stephen Hamer, Fabio M. V. Rossi

**Affiliations:** ^1^ School of Biomedical Engineering, University of British Columbia, Vancouver, BC, Canada; ^2^ Department of Medical Genetics, University of British Columbia, Vancouver, BC, Canada

**Keywords:** myogenic progenitor cells, muscle regeneration, cell therapeutic, synthetic biology (synbio), muscle stem cell (MuSC), iPSCs (induced pluripotent stem cells)

## Abstract

The generation of myogenic progenitors from iPSCs (iMPs) with therapeutic potential for *in vivo* tissue regeneration has long been a goal in the skeletal muscle community. Today, protocols enable the production of potent, albeit immature, iMPs that resemble Pax7+ adult muscle stem cells. While muscular dystrophies are often the primary therapeutic target for these cells, an underexplored application is their use in treating traumatic muscle injuries. Notably absent from recent reviews on iMPs is the concept of engineering these cells to perform functions post-transplantation that non-transgenic cells cannot. Here, we highlight protocols to enhance the generation, purification, and maturation of iMPs, and introduce the idea of engineering these cells to perform functions beyond their normal capacities, envisioning novel therapeutic applications.

## 1 Introduction

Life-long maintenance of skeletal muscle integrity is maintained by a population of tissue resident adult muscle stem cells (MuSCs), or “satellite cells”, embedded between the extracellular matrix (ECM) and individual myofibers (Mauro, 1961). The indispensable role of MuSCs, distinguished by their tissue-specific expression of the transcription factor Pax7, has been repeatedly demonstrated (Lepper et al., 2011; Murphy et al., 2011; Relaix and Zammit, 2012; Von Maltzahn et al., 2013). Despite protocols that afford efficient isolation of primary MuSCs from both human and murine tissues, therapeutic application of these cells is severely limited by donor availability and an inability to expand these cells *in vitro* (Montarras et al., 2005; Yi and Rossi, 2011; Liu et al., 2015; Jensen et al., 2021). Pluripotent stem cells (PSCs) have emerged as a promising, scalable, source of myogenic progenitors (MPs). Studies into the developmental origins of MuSCs have led to a deep understanding of the biological processes underlying the emergence of these cells within skeletal muscle tissue (Chal and Pourquié, 2017). These efforts, along with those affording the generation of PSCs via reprogramming of adult somatic tissue (iPSCs), have been foundational to the concept of using autologous MPs therapeutically (Takahashi and Yamanaka, 2006; Barberi et al., 2007).

In this perspective, we present the state-of-the-art in generating and purifying therapeutically relevant iPSC-derived MPs (iMPs), focusing on workflows that enhance their similarity to MuSCs and improve engraftment for *de novo* muscle formation and MuSC niche repopulation (Table 1). We then explore an emerging area of cellular engineering which, despite rapid adoption in fields like immunology, remains relatively undeveloped in skeletal muscle therapeutics. Synthetic biology and the engineering of cells to perform functions beyond their natural roles presents a transformative opportunity to reshape the current paradigm of iMP-based therapies.

**TABLE 1:** Key findings published that have progressed the field of iMP generation to its current state. The stability and flexibility afforded by directed differentiation are highlighted along with the maturation characteristics that we believe to be critical to improving iMP therapeutic application.

Reference	Differentiation technique/key advancement	*in vitro* iMP validation	*in vivo* iMP engraftment	Purification strategy	Maturity assessment?
Awaya et al. (2012)	First example of directed differentiation of human iPSCs into iMPs	iMP Pax7 expression (RT-PCR, IF) iMP-derived myoblasts/myofibers express of MyoG, MYH, and Dystrophin (IF)	Yes – CTX injury to TA transplanted with 1.0 × 10^5^ - 5.0 × 10^5^ iMPs. Pax7+ cells and hLaminin myofibers observed 24 w.p.i	Crude replating of embryoid body outgrowths at low density	No direct assessment of iMP maturation state but reinjury following *in vivo* iMP transplantation show Pax7+ cells capable of activation and self-renewal
Darabi et al. (2012)	First example of direct reprogramming of iPSCs into iMPs via inducible Pax7 overexpression; rescue of dystrophin expression	iMP Pax7 expression (qPCR, IF) iMP-derived myoblasts/myofibers express MyoG, MYH (IF)	Yes – CTX injury to TA transplanted with 3.0 × 10^5^ iMPs. Pax7+ cells and hDystrophin+ myofibers observed 8 w.p.i	Cell sorting of Pax7-GFP+ reporter cells	Improvements to absolute and specific force production capacity in treated legs observed but no assessment of Pax7+ cell maturity post-engraftment
Tedesco et al. (2012)	First example of direct reprogramming of iMPs using MyoD; iMPs engineered to express SGCA and rescue LGMD2D phenotype	iMP MyoD expression (IF, RT-PCR) iMP-derived myoblasts/myofiber express MyoG, MYH (IF, RT-PCR) and SGCA (IF)	Yes - Intramuscular SGCA expression following transplantation with continued tamoxifen administration	Cell sorting of SSEA1- cells	n/a
Chal et al. (2015), Chal et al. (2016)	Standardization of a defined directed differentiation protocol using serum free conditions	MyoG-driven reporter to track myogenic differentiation Expression of Pax3, Pax7, MyoG, MyoD, and MYH corresponding to developmental stage during differentiation (IF)	Yes – 1.0 × 10^5^ cells transplanted into uninjured mdx mice restored dystrophin expression	Cell sorting of Pax7-GFP+ reporter cells	No direct assessment of iMP maturation state but histological analysis post-transplantation show mature hDystrophin + fibers with hPax7 + cells within the MuSC niche
Hicks et al. (2018)	Directed differentiation followed by Erbb3+/NGFR+/CD57- FACS-purification improves engraftment of CRiSPR–Cas9 edited cells	Sorted iMPs express Pax7 and MyoD (IF, qPCR); MYF5, MyoG (qPCR) Improved iMP-derived myofiber maturity MYH (IF)	Yes - 1.0–2.0 × 10^6^ CRiSPR–Cas9 cells, edited to retore dystrophin expression, transplanted into mdx mice restored dystrophin expression with significant number of iMP-derived myofibers observed	Cell sorting on ERBB3+, NGFR+, CD57- iMPs	Engrafted iMPs performed better *in vivo* compared to cultured fetal MPs but were less potent than transplants using freshly isolated fetal MPs
Xi et al. (2020)	Generation of human skeletal muscle atlas dataset and maturation state comparison to iMPs generated by directed differentiation	scRNA-seq comparing myogenic potential of 3 commonly referenced directed differentiation protocols to *in vivo* human developmental data	n/a	Cell sorting on ERBB3+, NGFR+, CD57- iMPs	In-depth transcriptomic analysis (scRNA-seq) of iMPs demonstrate that these cells do not mature beyond the embryonic-to-fetal stage observed in human development
Mashinchian et al. (2022)	Directed differentiation of iMPs in 3D co-culture system alongside embryonic endothelial cells and fibroblasts	Sorted iMPs express Pax7 (IF) Sorted iMP-derived myoblasts/myofibers express MyoG, MyoD, and MYH (IF)	Yes – CTX injury to TA of MDX mouse transplanted with 2.5 × 10^4^ sorted iMPs restored dystrophin expression, improved eccentric force production, and displayed capacity to activate following reinjury	Cell Sorting on CD56 +, ITGA9+ cells correspond with Pax7-GFP reporter	Freshly isolated embryonic skeletal muscle progenitors and iMPs were transplanted and engraftment efficiency compared, demonstrating improved iMP-derive myofiber maturation
Mavrommatis et al. (2023)	Directed differentiation of iMP organoids	In-depth characterization of iMP development and maturation state within organoids (IF, scRNA-seq)	Yes - CTX injury to TA transplanted with 1.0 × 10^5^ sorted iMPs. Engraftment claimed but not quantified or characterized	Cell sorting on CD82 + cells	In-depth transcriptomic analysis (scRNA-seq) of iMPs within organoids a correlate with those present at human developmental week 17–18

## 2 Making muscle: differentiation and purification of iMPs

**FIGURE 1:**
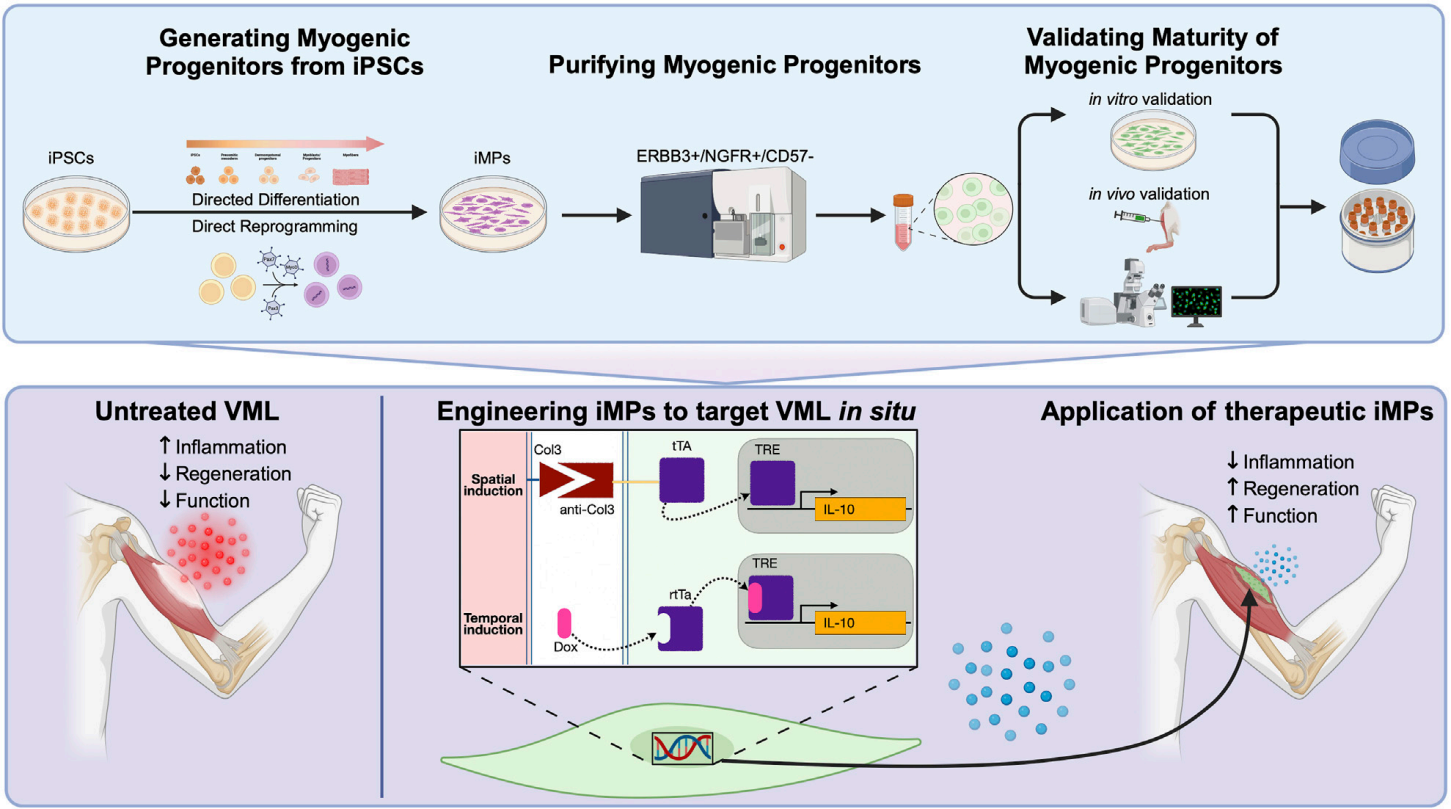
(Top panel) Standardized workflow for the generation and validation of iPSC-derived myogenic progenitors (iMPs) utilizing either directed differentiation or direct reprogramming approaches. Following differentiation, FACS isolation using ERBB3+/NGFR+/CD57− result in iMPs that are more mature, potent, and phenotypically comparable to MuSCs. Verification of myogenicity immediately after cell sorting include immunofluorescent staining for markers like Pax7 and MyoG and MYH and Dystrophin after secondary differentiation into myotubes. In parallel, cells should be further validated for their capacity to engraft and form *de novo* myofibers *in vivo*. Further histological assessment should confirm the capacity for some iMPs to retain Pax7 expression and populate the MuSC niche. (Bottom panel) An example of iMPs engineering to target volumetric muscle loss (VML) pathophysiology. Untreated VML is underscored by dysregulated fibrosis in the damaged muscle leading to poor regenerative potential. Engineering iMPs that express IL-10, a cytokine demonstrated to have potent pro-regenerative effects in VML, could be further enhanced by the spatio-temporal delivery afforded by this system. The improved regenerative environment should act synergistically to alleviate fibrosis and improve myogenic potential in the transplanted cells.

Today, a number of well-established protocols for the production of iMPs exist, each falling into one of two categories (Iberite et al., 2022). Direct reprogramming relies on the overexpression of myogenic transgenes to force myogenic potential, while directed differentiation generates iMPs through meticulous recapitulation of the conditions that lead to developmental emergence of MuSCs *in vivo* (Hicks et al., 2018; Xi et al., 2020; Nalbandian et al., 2021). While these two methods for generating iMPs differ, both aim to produce cells that can be used directly or, preferably, cryopreserved in viable cell banks for downstream application (Figure 1).

### 2.1 Direct reprogramming

Overexpression of the myogenic transcription factors MyoD and Pax7 both reliably induce iPSCs toward a myogenic fate. The first examples of iMP direct reprogramming demonstrated that activation of these inducible transgenes generated cells that reliably engraft into muscle tissue, and are capable of restoring tissue specific expression of dystrophin and a-sarcoglycan in their respective murine disease models (Darabi et al., 2012; Tedesco et al., 2012). Since these initial demonstrations, direct reprogramming protocols have been used to reliably produce iMPs with similar regenerative capacity (Tanaka et al., 2013; Quattrocelli et al., 2015; Uezumi et al., 2016; Azzag et al., 2022). Despite these successes, a number of caveats specific to direct reprogramming warrant consideration. First, many direct reprogramming strategies adopt the use of small molecules and growth factors to prime iPSCs towards a mesodermal lineage prior to myogenic transgene induction, raising the question of whether introducing transgenes for the final step of myogenic specification is truly necessary (Magli et al., 2017; Rao et al., 2018; Selvaraj et al., 2019). Correspondingly, direct reprogramming inherently skips critical developmental maturation steps and little has been done to assess the relative developmental maturity of iMPs generated using this method. We will elaborate on the importance of iMP maturity in later sections; however, the lack of evidence for maturation in these cells represents one of the most critical shortcomings of direct reprogramming. Concerns over the risks associated with random transgene integration in direct reprogramming have also drawn scrutiny. Targeting transgenes to genetic safe-harbor loci and transient overexpression systems have been developed to reduce the perceived risks associated with transgene integration but are likely precautionary and immaterial to therapeutic application (Sadelain et al., 2012; Ordovás et al., 2015; Kim et al., 2017; Kim et al., 2021; Shin et al., 2020).

Despite the drawbacks associated with direct reprogramming the FDA has recently given clinical trial approval for a first-of-its-kind muscular dystrophy treatment in which allogeneic cell banks, induced to a myogenic lineage using Pax7 overexpression, will be assessed for safety, tolerability, and engraftment capacity in DMD patients (Myogenica, 2024). Successes in these early trials have the potential to transform the landscape of myopathy-targeting therapeutics and open the door to more advanced therapeutic modalities, as we discuss below.

### 2.2 Directed differentiation

Unlike direct reprogramming, directed differentiations use transgene-free methods of generating iMPs, relying on a holistic consideration of the developmental conditions that give rise to adult MuSCs. The first directed differentiation protocols were adapted from human embryonic stem cell (hESC) systems, and relied on undefined medias to differentiate embryoid body outgrowths into iMPs (Barberi et al., 2007; Chang et al., 2009; Mizuno et al., 2010; Awaya et al., 2012). While heterogeneity in the resulting populations was duly noted, these early studies provided evidence of myogenic potential in human iPSCs, paving way for more sophisticated protocols.

In 2016, two seminal papers established the culture conditions for generating Pax7+ myogenic progenitors from human iPSCs, optimizing an approach that induces presomitic mesoderm (PSM) specification prior to myogenic progenitor differentiation (Chal et al., 2015; Chal et al., 2016; Shelton et al., 2016). The resulting iMPs demonstrated robust myogenic capacity, but the rationale for the timing and concentration of certain growth factors remains dubious, even today. As these remain two of the most cited protocols for the generation of iMPs, a more granular screening aimed at optimizing these conditions is warranted and overdue. Data on the transition from early fetal to adult skeletal muscle in human tissue isolates are publicly available and may provide insights that would resolve this ambiguity (Xi et al., 2020).

Today, generation of iMPs using PSM to myogenic precursor differentiation has become the gold-standard in directed differentiations. However, the resulting cells are heterogeneous and there remains potential for further optimization and refinement (Bou Akar et al., 2024). Directed differentiations can generate iMPs that stably express Pax7 and are a more developmentally accurate recapitulation of MuSC-like cells than those generated via direct reprogramming. Standardization of directed differentiation protocols will be crucial to delivering therapeutic-grade iMPs. Towards this end STEMCELL technologies has released a product (STEMdiff™ Myogenic Progenitor Supplement Kit), that reliably produces a robust population of iMPs. cGMP qualification of products like this would represent a major step towards therapeutic application.

### 2.3 Purification strategies to isolate iMPs

Regardless of the method used to generate iMPs, their therapeutic potential remains constrained by population heterogeneity. Direct reprogramming protocols utilize Pax7-driven fluorescent reporters to facilitate iMP purification, but neural crest progenitors, which also express Pax7, commonly contaminate these cultures (Basch et al., 2006). Non-transgenic directed differentiation strategies lack fluorescent reporters, necessitating the identification of surface markers that correlate with myogenic Pax7 expression. Cell surface markers for the purification of adult MuSCs have been identified, but are not reliably expressed in human iMPs (Wosczyna and Rando, 2018; Mierzejewski et al., 2020). Characterization of Pax7+ iMPs has led to the identification of several combinations of cell surface markers that aid in their purification. While CD54, CD10, CD82, CD56, and CDH13, and FGFR-4 all correlate with Pax7+ expression in human iMPs, the epidermal growth factor receptor ERBB3 has thus far been demonstrated the most reliable (Choi et al., 2016; Uezumi et al., 2016; Hicks et al., 2018; Sakai-Takemura et al., 2018; Wu et al., 2018; Nalbandian et al., 2021). In combination with positive selection for CD271 and negative selection of CD57 neuroectodermal contaminates, ERBB3 allows robust purification of iMPs that are phenotypically similar to the MuSCs observed developmentally during secondary myogenesis (Hicks et al., 2018; Tey et al., 2019). To validate myogenicity after cell sorting, iMPs should be plated and immediately stained for Pax7 and MyoG. Additional confirmation of myogenic potential should be confirmed by staining for myosin heavy chain (MYH) and dystrophin following subsequent differentiation into myotubes.

## 3 Maturation of iPSC derived myogenic progenitors beyond the fetal phenotype

Despite improvements in the generation and purification of iMPs over the last decade, these cells invariably retain a late-embryonic/early-fetal phenotype, which like fetal MuSCs, show reduced engraftment capacity compared to adult MuSCs (Castiglioni et al., 2014; Xi et al., 2020). Strategies to address this lack of maturity include culturing iMPs in a 3D macroenvironment, external stimulation, and *in vivo* maturation (Khodabukus et al., 2019; Selvaraj et al., 2019; Kim et al., 2020; Sun et al., 2022; van der Wal et al., 2023; Crist et al., 2024). As maturation state remains the most significant identifiable cause of poor iMP engraftment, a collective effort must be made to address this problem.

### 3.1 3-dimensional and co-culture systems

3D directed differentiation of iMPs attempts to integrate the culture conditions established in 2D protocols with the spatial/environmental cues afforded by 3D organization. In one study, fully humanized multilineage embryoids containing iPSCs, growth-arrested embryonic fibroblasts and embryonic endothelial cells were grown together using a directed differentiation protocol, resulting in robust myogenic induction of the iPSCs; after only 13 days 40%–50% of cells in these embryoids were Pax7+ (Mashinchian et al., 2022). Unfortunately, the method used in this study to quantify Pax7 via RNA-FISH misrepresents the true myogenic Pax7 population, and staining with surface markers for Pax7-associated proteins significantly reduced the number of truly myogenic cells. In addition, despite acceptable engraftment and improvements to functional force production, these cells exhibited an embryonic (week 9) phenotype, indicating that they are no more mature than those produced in 2D systems.

In a more recent publication, human skeletal muscle organoids formed a largely myogenic population of cells (∼90%). Interestingly, a significant number of mesenchymal (∼4%) and neural (∼5%) cells also populated these 3D structures. While not explicitly studied, the heterogenous 3D structure was likely partially responsible for the maturation of the resulting Pax7+ iMPs, which closely resembled a fetal week 17 phenotype (Mavrommatis et al., 2023). Cell-cell communication analysis of the scRNA sequencing data generated in this work identified a number of upregulated ECM components (COL1A2, COL5A2, COL5A3, FN1) and transcription factors (FBN1, CHODL, SPRY1*)* that could be targeted in 2D or 3D systems to further mature iMPs.

While inducing differentiation of iMPs using 3D culture systems is relatively new, the application of differentiated iMPs within 3D systems has been widely reported (Rao et al., 2018; Selvaraj et al., 2019; van der Wal et al., 2023). It is established that 3D co-cultures can mimic the MuSC niche, enhance myofiber maturation, boost *in vivo* engraftment potential, and act as effective disease models (Quarta et al., 2016; Quarta et al., 20017; Bersini et al., 2018; Maffioletti et al., 2018; Rao et al., 2018; Bakooshli et al., 2019; Kim et al., 2020; Urciuolo et al., 2020; Massih et al., 2023). While recovery of mature iMPs from 3D systems may limit certain therapeutic applications (e.g., those requiring single cell suspensions), it may enhance their utility in others (e.g., traumatic skeletal muscle injury). 3D systems should be considered valuable tools for assessing iMP maturation and could help to further identify culture conditions that enhance this process.

### 3.2 Stimulation of iMPs to improve maturity

Stimulation of myogenic cells can be achieved through either mechanical, chemical, electrical, or static tension. Early studies of C2C12-derived myofibers seeded within 3D collagen scaffolds demonstrated that electrical stimulation of these cells improved sarcomere organization and myofiber maturity (Park et al., 2008). In 2018, Rao *et al.* demonstrated that myofibers grown within their 3D scaffolds supported a population of self-sustaining Pax7+ iMPs (Rao et al., 2018). Despite thorough characterization of this stimulatory effect on myofiber maturity and force production, the Pax7+ iMP population was not further characterized. In a more recent study, van der Wal *et al.* directly compared directly differentiated iMP-derived myofibers to those of primary human myoblasts, showing that electrical stimulation of the 3D scaffolds produced comparable force production measurements (van der Wal et al., 2023). While this study provided a detailed physical and proteomic analysis of the resulting myofibers, the presence and maturity of Pax7+ cells within the constructs were not reported. *In vivo,* maturation of the MuSC compartment coincides with myofiber maturation following secondary myogenesis. Future studies should make use of systems like those described above to assess the effects of stimulation on the capacity of iMP-derived myofibers to support maturation of the associated iMPs.

### 3.3 The body is the best bioreactor

Human MuSC development is a spatiotemporally coordinated process guided by concurrent maturation of the surrounding tissue (Chal and Pourquié, 2017). Differentiation of iPSCs to fully mature iMPs may therefore be limited by constraints inherent to culture systems. Comparing MuSCs with iMPs matured *in vivo* or *in vitro* has demonstrated that *in vivo*-matured iMPs more closely resembled adult MuSCs, whereas *in vitro*-matured iMPs retained a more fetal-like transcriptomic profile (Incitti et al., 2019). Important myogenic regulators (Stat3, Jun, Itga7, Tgfb2, Notch1, Notch3, and Jag1) and genes involved in ECM regulation were upregulated in the *in vivo* matured iMPs compared to those matured *in vitro.* Future studies aimed at targeted activation or inhibition of signaling pathways in which these factors are involved may improve maturation of iMPs *in vitro*. Likewise, incorporating the identified ECM modalities within engineered scaffolds may expedite the maturation of these cells when applied within certain therapeutic contexts. The key takeaway from these data is that iMP maturity *in vitro* may be less significant to successful therapeutic application than previously thought; instead, we may be able to rely on *in vivo* maturation to carry these cells towards a more MuSC-like phenotype.

## 4 Engineering iMPs to improve therapeutic potential

Advancements in the generation, purification, and maturation of iMPs justify consideration of how to proceed with their therapeutic application. Recent reviews have highlighted the need for pre-clinical testing of the safety and efficacy of iMPs but emphasis is often placed on those capable of addressing muscular dystrophies (Iberite et al., 2022; Sun et al., 2024). In this section we will instead discuss tissue engineering strategies focused on the treatment of volumetric muscle loss (VML). We will then consider how we might employ iMPs as a therapeutic platform, focusing potential application of these cells as treatments for systemic diseases.

### 4.1 Current therapeutic applications of iMPs in VML

VML is characterized by a loss of tissue architecture that leads to chronic fibrosis and an associated loss of function (Corona et al., 2015). Current clinical treatments rely on crude muscle flap surgeries, which are limited by donor-site availability (Greising et al., 2017). Experimental therapeutics targeting VML aim to restore tissue integrity and function through the use of 3D scaffolds seeded with myoblasts or MuSCs (Corona et al., 2013; Quarta et al., 2017; Goldman et al., 2018). Despite the obvious application of iMPs as a substitute for patient-derived MuSCs, few studies have reported successful therapeutic application of these cells (Wu et al., 2021; Pinton et al., 2023). iMP maturity likely dictates survival within the scaffold and the capacity to successfully generate functional *de novo* muscle.

### 4.2 Augmenting scaffold design to improve iMP efficacy

Scaffold design is critical to targeting VML pathophysiology (Wolf et al., 2015). In previous sections we have alluded to different ECM components that might help to mature iMPs *in vitro*. Current scaffolds can include either synthetic or natural material compositions, with natural hydrogels generally favored due to their intrinsic biocompatibility (Fischer et al., 2020; Luo et al., 2022). Hydrogels are frequently generated using proteins like collagen or fibrinogen, or polysaccharides such as hyaluronic acid or alginate. Those composed of proteins - specifically collagen - rarely consider particular isoforms, which have diverse functional characteristics (Goldman et al., 2018; Matthias et al., 2018; Pollot et al., 2018). Future studies should aim to incorporate specific collagen isotypes, like those already identified as important for iMP maturation, into scaffolds seeded with iMPs to improve maturation and survivability (Incitti et al., 2019). Additional considerations, such as the addition of growth factors and materials that promote revascularization and reinnervation must also be considered but are outside the scope of this perspective (Wu et al., 2021).

### 4.3 iMPs as *in vivo* biologics factories

Skeletal muscle is a highly vascularized, metabolically active tissue, making it an excellent vehicle for the delivery of biological therapeutics. The engraftment of iMPs, while imperfect, is likely sufficient to deliver effective cell-based synthetic gene products *in situ*. A key advantage of iMPs is their capacity to be extensively engineered prior to differentiation and/or transplantation, affording flexibility in tailoring treatments. Utilizing iMPs as a delivery platform for therapeutics like growth factors, cytokines, and biologics represents a highly promising, yet largely untapped, application. This approach could revolutionize the delivery of therapeutics by directly targeting affected tissues, enhancing treatment precision and efficacy.

Following VML, a dysregulated immune response results in persistent inflammation, elevated TGFβ expression within the damaged tissue, and resulting in fibrosis (Larouche et al., 2021). Experimental treatments have successfully alleviated fibrosis through systemic administration of anti-fibrotic agents that target TGFβ, but these treatments have dangerous off-target effects (Garg et al., 2014; Greising et al., 2019; Larouche et al., 2022). More recently, work has demonstrated that local administration of the pro-regenerative cytokine IL-10 can improve the regeneration in skeletal muscle following VML (Huynh et al., 2023). Engineering iMPs to target inflammation and fibrosis - either by locally secreting anti-TGFβ antibodies or by counteracting inflammation using pro-regenerative cytokines such as IL-10 - could enhance regeneration after VML while mitigating the risks of systemic anti-TGFβ therapies (Borok et al., 2020; Narasimhulu and Singla, 2023). The capacity to generate inducible transgenes, controlled either temporally or spatially, would further improve the precision of these treatments (Figure 1).

In another example, we imagine a system in which iMPs engineered to constitutively express and secrete hormones responsible for blood glucose-homeostasis, might alleviate hyperglycemia in diabetic patients (Xie and Fussenegger, 2018). Synthetic circuits enabling glucose-mediated insulin secretion have been developed, bypassing the need for islet cells, which remain targeted by the immune system in type 1 diabetics receiving islet transplants (Xie et al., 2016). Type 2 diabetes might also be targeted using iMP-delivered synthetic gene circuits that secrete adiponectin to improve insulin sensitivity (Ye et al., 2017). Hypothetically, these synthetic gene circuits, applied to iMPs transplanted in otherwise healthy diabetic patients, could counteract hyperglycemia without the documented shortcomings of iPSC-based pancreatic beta cell therapies (Maxwell and Millman, 2021).

Thoughtful design of inducible synthetic transgenes should allow for iMPs to deliver therapeutics either locally or systemically. While the immediate application of these systems to target VML pathology are evident, the application of iMPs as a therapeutic delivery platform targeting systemic disease has yet to be discussed in the literature. The capacity for iMPs to persist long term as engrafted myofibers makes them an attractive alternative to other cell-based therapies that are often limited by poor engraftment efficiency and survivability (Levy et al., 2020; Zhou et al., 2021). We propose that future studies aiming to address disease using cell therapeutics consider the use of iMPs as a reliable delivery platform.

### 4.4 iMPs as biosensors to study cellular dynamics

Temporal regulation of synthetic gene circuits is easily achieved through the use of inducible promoters, but the spatial organization of regenerating skeletal muscle may provide additional, therapeutically relevant, cues. Spatial transgene regulation has been achieved through the engineering of synthetic receptors capable of recognizing specific ligands, resulting in customizable sense/response behaviors (Roybal et al., 2016; Scheller et al., 2018). Synthetic notch (synNotch) receptors have proven a particularly powerful tool, capable of inducing the expression of myogenic transgenes in a spatially-regulated context (Morsut et al., 2016). Application of the synNotch system *in vivo* has demonstrated broad utility, from serving as an intercellular contact sensor capable of fate-mapping endothelial cells during development to enhancing CAR cell therapeutic efficacy (Hyrenius-Wittsten et al., 2021; Zhang et al., 2022). iMPs engineered to express customized synNotch constructs could provide spatial, context-dependent, regulation of transgene activation/deactivation. For example, pro-fibrotic collagens 3, 4 and 6 are significantly upregulated in the fibrotic lesion following VML (Hoffman et al., 2022). A self-regulating, cell-based synNotch system that uses antiCol3-tTa to control expression of anti-TGFβ antibody expression could enable targeted delivery, limiting expression to areas only where pro-fibrotic collagens are present (Figure 1).

### 4.5 Engineering iMPs to exogenously stimulate myofibers

When skeletal muscle is insufficiently innervated exogenous electrical stimulation can improve both the maturation and force production of the myofibers, aiding in the rehabilitation of muscle strength (Gordon and Mao, 1994; Crist et al., 2024). Optogenetic switches, which allow for transdermal stimulation of skeletal muscle using visible light, represents an attractive alternative to invasive patch clamps. Optogenetic switches have already proven capable of driving skeletal muscle contraction both *in vitro* and *in vivo* (Bruegmann et al., 2015; Ganji et al., 2021). Their therapeutic application as part of an iMP-based treatment should be investigated as it may allow for partial restoration of muscle function in spinal cord injury patients; or, at the very least, assist in the rehabilitation process.

## 5 Discussion/conclusion

The finding that myogenic differentiation is enhanced by prior PSM specification, whether it be in directed differentiation or direct reprograming protocols, has been fundamental to the generation of therapeutically relevant iMPs. Likewise, the ability to accurately purify the ERBB3+/NGFR+/CD57- myogenic Pax7+ population using cell sorting has considerably improved the purity of the resulting iMPs. Although imperfect, the current maturation capacity of these protocols allows for efficient engraftment of these cells and the generation of iMP-derived myofibers *in vivo*. While improvements within each of these categories are sure to further enhance the capacity for these cells to behave like adult MuSCs, using current iMPs as a therapeutic delivery platform opens the door to a wonderfully diverse and novel way of treating myopathies and systemic diseases alike. We encourage those working on cell-based therapies to consider new ways in which these cells can be employed and toward a future in which long-term iMP-based therapeutics can become a platform for therapeutics delivery.

## Data Availability

The original contributions presented in the study are included in the article/Supplementary Material, further inquiries can be directed to the corresponding author.
